# The Abundance of the *nifH* Gene Became Higher and the *nifH*-Containing Diazotrophic Bacterial Communities Changed During Primary Succession in the Hailuogou Glacier Chronosequence, China

**DOI:** 10.3389/fmicb.2021.672656

**Published:** 2021-05-31

**Authors:** Yingyan Wang, Yulan Chen, Qinyu Xue, Quanju Xiang, Ke Zhao, Xiumei Yu, Qiang Chen, Menggen Ma, Hao Jiang, Xiaoping Zhang, Petri Penttinen, Yunfu Gu

**Affiliations:** ^1^Department of Microbiology, College of Resource Sciences and Technology, Sichuan Agricultural University, Chengdu, China; ^2^Institute of Chengdu Mountain Hazards and Environment, Chinese Academy Sciences, Chengdu, China

**Keywords:** Hailuogou Glacier chronosequence, primary succession, N_2_-fixing microorganism, community composition, stochastic processes

## Abstract

Primary successional ecosystems and the related soil development are often N limited. To date, N_2_-fixing communities during primary succession in alpine ecosystems have remained underexplored. In this study, we applied quantitative PCR (qPCR) quantitation and targeted amplicon sequencing of *nifH* in the Hailuogou Glacier foreland to investigate the succession of N_2_-fixing communities in five sites along a 62-year chronosequence. The abundance of the *nifH* gene increased along the primary succession in the chronosequence and correlated positively with pH, acetylene reduction activity, and water, organic C, total and available N, and available P contents. The increases in alpha diversity along the chronosequence may have been partly due to less competition for resources. In contrast to the clear separation based on soil properties, the changes in the diazotrophic community composition lacked a clear trend and were associated mostly with changes in soil available K and organic C contents. The changes among differentially abundant genera were possibly due to the changes in plant coverage and species composition. The whole primary succession of the diazotrophic communities was consistent with stochastic community assembly, which is indicative of low competitive pressure.

## Introduction

The first steps of primary succession in recently exposed environments, for example, after glacier retreat, include colonization by pioneer communities and nutrient mobilization (Odum, 1969). Many alpine mountain glaciers have retreated at an accelerated rate in recent decades due to global warming (Li et al., 2011; Wang et al., 2011). The glacier retreat provides ideal models for exploring soil primary succession; the exposed, originally barren land goes through a succession of soil processes, including carbon (C) and nitrogen (N) accumulation, transformation, and nutrient cycling (Hopkins et al., 2007; Nemergut et al., 2007). As early colonizers, the soil microorganisms play an important role in mediating these processes. For example, in the newly exposed terrain with sparse vegetation, C fixation is carried out by the phototrophic cyanobacteria and algae (Frey et al., 2013). By focusing on the soil microorganisms, the period needed to decipher the community development patterns in primary succession will be considerably shorter than when studying plant or animal communities (Jessup et al., 2004).

Primary successional ecosystems and the related soil development are often N limited (Göransson et al., 2011). Plants cannot directly use dinitrogen gas (N_2_) and organic N, which make a large proportion of N in the environment. Only specialized microorganisms, diazotrophs, can fix N_2_ and mineralize organic compounds into ammonia. In many natural ecosystems, including soils exposed by glacier retreat, N_2_ fixation is the major N source (Yeager et al., 2004; Nemergut et al., 2007; Abed et al., 2010; Göransson et al., 2011). Nitrogenase, the N_2_-fixing enzyme complex, is evolutionarily conserved, and most of the N_2_-fixing microorganisms carry *nifH*, which encodes one of the components of nitrogenase (Zehr et al., 2003), making *nifH* an ideal marker to analyze the succession of N_2_-fixing communities. Using *nifH* as a marker, Brankatschk et al. (2011) found that along a chronosequence in a glacial forefield, the abundance of *nifH* was higher in a 50-year successional age site than in a 10-year successional age site. Similarly, along a 44-year chronosequence in a glacial forefield, the abundance of *nifH* increased with successional age, and the diazotrophic community compositions, analyzed based on one sample per successional age, were slightly different (Zeng et al., 2016). In a metagenomics analysis, the *nifH* sequences in Arctic glacier forefield soils were related most closely to Alphaproteobacteria, Betaproteobacteria, Cyanobacteria, and Firmicutes (Nash et al., 2018). Julien et al. (2011) concluded that in the initial stage, Cyanobacteria increase soil N content and stabilize the soil by forming soil crusts; as the N content allows the growth of plants, cyanobacteria are displaced. However, Duc et al. (2009) noticed that Firmicutes and Cyanobacteria were abundant in an 8-year and a 70-year ice-free forefield, respectively.

The glaciers in Gongga Mountain, China, are melting at an accelerated rate with global warming (Zhou et al., 2013; Lei et al., 2015). Among the numerous glacier forelands of Gongga Mountain, the Hailuogou Glacier has retreated continuously since the end of the Little Ice Age (Li et al., 2011), resulting in a distinct soil and primary vegetation succession chronosequence in the glacier forefield. Previous studies have addressed soil development, succession of plant, bacterial and fungal communities, and soil phosphorus speciation and transformation and the related *phoD*-harboring bacterial communities along the chronosequence (Zhou et al., 2013, 2019; Yang et al., 2014; Jiang et al., 2018; Bai et al., 2020). To date, N_2_-fixing communities during primary succession in alpine ecosystems have remained underexplored. In this study, our aims were to analyze N_2_-fixing communities and changes in them during primary succession, to identify the specific taxa that characterize the changes, and to determine soil parameters associated with these changes. Using soil samples collected by Bai et al. (2020) from soils ice free for <5, 22, 40, 54, and 62 years, we applied quantitative PCR (qPCR) quantitation and targeted amplicon sequencing of *nifH* in the Hailuogou Glacier foreland to investigate the succession of N_2_-fixing communities along a 62-year chronosequence.

## Materials and Methods

### Study Area

The study area is as described by Bai et al. (2020). Briefly, Gongga Mountain, Sichuan, China, is on the southeastern edge of the Tibetan Plateau, with the highest peak at 7,556 m above sea level (ASL). The rate of retreat of the Hailuogou Glacier (29°34′07.83″N, 101°59′40.74″E) on the eastern slope of Gongga Mountain has accelerated during the last 100 years. The Hailuogou Glacier chronosequence area is described in detail by Zhou et al. (2013) and Lei et al. (2015). The area is at 2,855 to 2,982 m ASL, the average annual temperature is 4.2°C, and the average annual precipitation is 1,947 mm. Biotite schist, granodiorite and quartzite, and to a lesser extent phyllite, slate, and chlorite schist are the soil parent materials (Zhou et al., 2013).

### Soil Sampling

We analyzed soil samples from five sites along the chronosequence, collected by Bai et al. (2020) (Supplementary Figure 1 and Supplementary Table 1). The approximate successional age of the sites was defined based on a previous study (Jiang et al., 2018). The sites were as follows: BJ (T1 in Bai et al., 2020), succession time less than 5 years, bare rock with scattered glacial debris; T0 (T2 in Bai et al., 2020), succession time less than 22 years, no vegetation, a hailstone deposit in which the main material is gravel and hail; T1 (T3 in Bai et al., 2020), succession time approximately 40 years, horizontal distance from the glacier approximately 1 km, *Astragalus membranaceus*–*Hippophae rhamnoides*–willow vegetation; T2 (T4 in Bai et al., 2020), succession time approximately 54 years, horizontal distance from the glacier approximately 1.3 km, willow–*H. rhamnoides*–*Populus purdomii* vegetation; and T3 (T5 in Bai et al., 2020), succession time approximately 62 years, horizontal distance from the glacier approximately 1.5 km, *P. purdomii*–*Betula utilis*–*Abies fabri* vegetation.

As described by Bai et al. (2020), six sampling plots per site with a minimum distance of 100 m from each other were selected. To avoid the effects of erosion and deposition, all sampling pits were in the middle of gentle slopes, far from the small streams. Five soil samples per plot were collected using a 6.5-cm-diameter steel push corer from the top 20 cm and mixed into one approximately 1.0 kg homogenized composite sample. Visible plant roots and residues were removed before mixing. Soil samples were stored in plastic bags on ice and divided into two portions: one was air-dried and sieved through a 2-mm mesh screen for physicochemical analyses, and the other was stored at -80°C for DNA extraction.

### Physicochemical and Nitrogenase Activity Analyses

The soil pH was determined (AB15 pH meter, Accumet, Fisher Scientific) at a soil-to-deionized water ratio of 1:2.5 (w/v). The contents of soil organic carbon (SOC) and total nitrogen (TN) in dried soil ground with a ball mill (Retsch PM 200 Planetary Ball Mill, Haan, Germany) were determined by combustion (CNS-2000, LECO, St. Joseph, MI, United States). Available phosphorus (AP) was extracted with 0.5 M NaHCO_3_ (pH 8.5) at a soil-to-solution ratio of 1:20 (w/v) for 30 min and measured by a colorimetric procedure. Available potassium (AK) was extracted with 1 M CH_3_COONH_4_ (pH 7.0) at a soil-to-solution ratio of 1:10 (w/v) for 30 min and determined by flame atomic absorption spectrometry. Available nitrogen (AN) was determined by the alkali hydrolysis diffusion method (Supplementary Table 2). The water content (WC) and electrical conductivity (EC) were measured by standard methods. Soil temperature (ST) at 10-cm depth was measured using a mercury-in-glass geothermometer with bent stems (Hongxing Thermal Instruments, Wuqiang, Hebei, China) at 13:00 on the soil sampling day. Soil nitrogenase activity was determined using the acetylene reduction assay method; ethylene concentrations were measured using a gas chromatograph with a flame-ionization detector (Perkin Elmer, Waltham, MA, United States) (Christophe et al., 2010).

### DNA Extraction

DNA was extracted from 0.5 g fresh soil using a FastDNA SPIN Kit for Soil (MP Biomedicals, Solon, OH, United States) according to the manufacturer’s instructions. The concentration and quality of extracted DNA were measured with a Nano-200 spectrophotometer (Aosheng, Hangzhou, China). DNA samples were stored at –20°C for further analysis.

### Quantification of *nifH*

The *nifH* gene abundance was quantified by qPCR with six technical replicates per sample using an ABI7500 sequence detection system (Applied Biosystems, New York, NY, United States). Standard curves to estimate *nifH* gene abundance consisted of a 10-fold serial dilution of a plasmid containing the *nifH* gene fragments (360 bp). The *nifH* fragments were amplified with primers PolF (5′-TGCGAYCCSAARGCBGACTC-3′) and PolR (5′-ATSGCC-ATCATYTCRCCGGA-3′) (Poly et al., 2001). Quantification was done in a reaction mixture containing 12.5 μl of ABI Power SYBR Green qPCR Master Mix (Applied Biosystems, New York, NY, United States), 1 μM of each primer, 1.25 μl of 20 ng μl^–1^ template DNA, and sterile distilled water to make up a final volume of 25 μl. The qPCR program consisted of an initial denaturation at 94°C for 8 min and 35 cycles of 94°C for 15 s, 55°C for 50 s, and 72°C for 30 s. The efficiency of *nifH* amplification ranged from 98% with an *R*^2^ of 0.9928.

### Preparation of *nifH* Amplicon Library and Sequencing

An internal fragment (360 bp) of the *nifH* gene was amplified with degenerated universal primers PolF and PolR (Poly et al., 2001). The reverse primer included a sequencing adapter and barcode sequences for multiplexing (Walters et al., 2011). Primers were synthesized at Sangon Biotech (Shanghai, China). PCR amplification was performed in triplicate with an ABI GeneAmp^®^ 9700 PCR System in a 50-μl PCR reaction mixture consisting of 5 μl of 10 × Ex Taq Buffer, 2 μl of dNTP mix (2.5 mM each), 0.5 μl of each primer (10 μM), 5 μl of 20 ng μl^–1^ template DNA, 31.5 μl of double-distilled water (ddH_2_O), and 2.5 U of Taq DNA polymerase (TAKARA, Otsu, Shiga, Japan). The PCR program consisted of an initial denaturation at 94°C for 4 min; 32 cycles of 20 s at 94°C, 20 s at 55°C, and 30 s at 72°C; and a final extension at 72°C for 10 min.

The triplicate PCR products were pooled, purified with a PCR Clean-up Purification Kit (MP Biomedicals, CA, United States), and quantified using a Qubit 2.0 fluorometer (Invitrogen, Carlsbad, CA, United States). Purified amplicons were pooled in equimolar concentrations and sequenced using a NEB Next^®^ Ultra^TM^ DNA Library Prep Kit for Illumina HiSeq (BioLabs Inc., United States). The raw sequence data were deposited in the NCBI Sequence Read Archive^1^ under accession number PRJNA511567.

### Sequence Data Analyses

After sequencing, *nifH* reads were filtered using the QIIME pipeline^2^ (Caporaso et al., 2010). Low-quality sequences with a quality score < 20, ambiguous nucleotides, or no matches to the primer and barcode were removed. The remaining sequences were converted to amino acid sequences using the FunGene Pipeline of the Ribosomal Database Project (Fish et al., 2013). Sequences whose translated proteins did not match the *nifH* protein sequence or that contained termination codons were discarded. The remaining sequences were aligned against the *nifH* gene database (Gaby and Buckley, 2014), and both non-aligned and chimeric sequences were removed. The remaining high-quality sequences were clustered into operational taxonomic units (OTUs) with UCLUST (Edgar, 2013) running in *de novo* mode at 95% amino acid similarity, and all singleton OTUs were removed.

### Statistical Analyses

Differences in soil properties were tested using a random permutation test (Zhang, 2014) and visualized using principal component analysis (PCA). Alpha diversity indices were calculated using the QIIME pipeline. Differences in alpha diversity were tested using the Kruskal–Wallis test, and Spearman correlations between alpha diversity and soil properties were calculated using the R package “vegan” in R v3.5.1 (R Core Team, 2017; Oksanen, 2018). Beta diversity was calculated as Bray–Curtis dissimilarities between sites using the R package “vegan” in R v3.5.1. Based on Bray–Curtis dissimilarities, variation in the diazotrophic communities was visualized using principal coordinate analysis (PCoA), followed by canonical analysis of principal coordinates (CAP) to estimate the contribution of edaphic factors to differences in the diazotrophic communities. Effect significance of factors was calculated using the permutest function with a maximum of 500 permutations over the CAP model. Based on distance matrix, the non-linear regression analysis was visualized using the R package “vegan” in R v3.5.1 (R Core Team, 2017). The relationships between edaphic properties and the diazotrophic community composition were tested using Mantel and partial Mantel tests. Data were analyzed and visualized using the R packages “vegan” and “ggplot2” in R v3.5.1 (Ito and Murphy, 2013; R Core Team, 2017; Oksanen, 2018).

The unweighted and weighted UniFrac (Lozupone and Knight, 2005) and mean nearest taxon distance metric were calculated using the R package “picante” (Kembel et al., 2010), followed by calculating the β-nearest taxon index (βNTI) using a null model approach (Stegen et al., 2012; Wang et al., 2013). βNTI values over 2 and below -2 indicate more and less than expected phylogenetic turnover, respectively, which suggest variable and homogeneous selection of deterministic processes, respectively; the differences in phylogenetic composition are considered to result from stochastic processes at | βNTI| < 2.

Differential abundance analysis at the genus level was done using the R package “DESeq2” (Love et al., 2014). Enriched genera (eGENs) and depleted genera (dGENs) were defined as genera with a difference in relative abundances (*P* < 0.1) in two contiguous sites along the chronosequence.

## Results

### Soil Properties, Acetylene Reduction Rate, and *nifH* Gene Abundance

The nutrient contents were as measured by Bai et al. (2020) (Supplementary Table 2). Briefly, WC increased gradually from the BJ site (successional age < 5 years) to the T3 site (successional age ≈ 62 years) (*P* < 0.05). EC was lowest in BJ and T0 and highest in T3 (*P* < 0.05). ST was lowest in BJ and highest in T0 (successional age ≤ 22 years), T2 (successional age ≈ 54 years) and T3 (*P* < 0.05) (Table 1). Soil acetylene reduction rates (ARA) ranged from 0.079 to 1.390 nmol C_2_H_2_ cm^–2^ h^–1^, being lowest in BJ and highest in T2 and T3 (*P* < 0.05) (Table 1). ARA correlated negatively with pH and positively with the other physicochemical factors (*P* < 0.05) (Supplementary Table 3).

**TABLE 1:** Some soil properties and acetylene reduction rate at the sampling sites along the Hailuogou Glacier chronosequence.

	BJ	T0	T1	T2	T3
Ph	7.82 ± 0.10a	7.12 ± 0.06b	7.33 ± 0.07b	6.80 ± 0.11c	6.23 ± 0.08d
WC (%)	0.092 ± 0.00d	0.159 ± 0.00c	0.155 ± 0.00c	0.193 ± 0.01b	0.227 ± 0.01a
EC (μS cm^–1^)	46.8 ± 0.84e	58.8 ± 1.22d	77.9 ± 0.53c	89.8 ± 0.55b	121.6 ± 4.50a
ST (°C)	-3.01 ± 0.37c	7.37 ± 0.24a	6.53c ± 0.26b	7.59 ± 0.18a	7.76c ± 0.21a
ARA (nmol C_2_H_2_ cm^–2^ h^–1^)	0.079 ± 0.01c	0.915 ± 0.03b	1.01 ± 0.02b	1.39 ± 0.14a	1.30 ± 0.03a

*Values (mean ± SE, n = 6) followed by different letters indicate statistically significant differences between sites (Tukey’s HSD test, P < 0.05). WC, water content; EC, electrical conductivity; ST, soil temperature; ARA, acetylene reduction rate; BJ, successional age < 5 years; T0, 22 years; T1, 40 years; T2, 54 years; T3, 62 years.*

**FIGURE 1:**
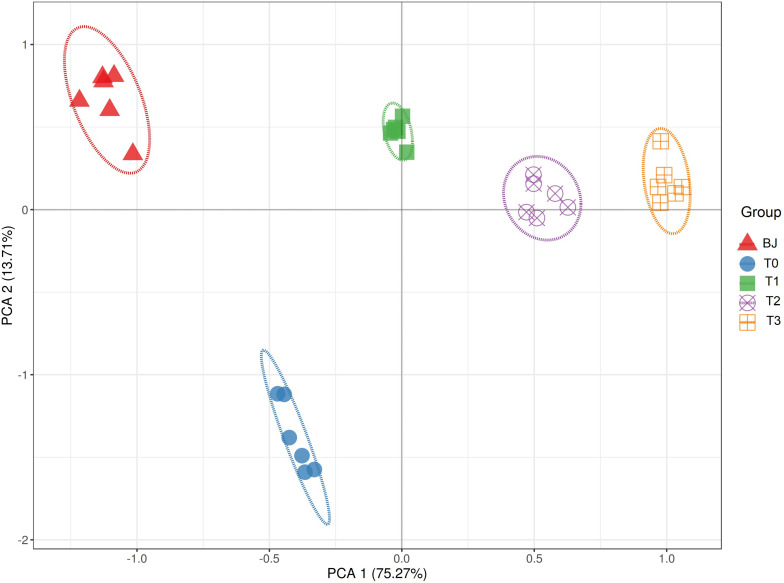
Principal component analysis (PCA) of the soil properties in the Hailuogou Glacier chronosequence. BJ, successional age < 5 years; T0, successional age 22 years; T1, successional age 40 years; T2, successional age 54 years; T3, successional age 62 years.

Based on the soil properties measured by Bai et al. (2020) and ST and ARA measured in this study, the sites were clearly separated according to the successional age along principal component 1, which explained over 75% of variation in the PCA, indicating that the primary succession in the Hailuogou Glacier chronosequence had resulted in distinct habitats (Figure 1).

The *nifH* copy numbers in the soils ranged from 3.641 log_10_ per gram dry soil in the BJ site to 7.703 log_10_ per gram dry soil in the soils from T3 site (Supplementary Figure 2), implying that the abundance of diazotrophic bacteria increased along the primary succession in the Hailuogou Glacier chronosequence. The nifH gene abundance correlated positively with DNA content, pH, ARA, WC, SOC, TN, AN, AP contents (*P* < 0.05), and negatively with pH (*P* < 0.01) (Supplementary Table 3).

### Relative Abundance of Major Phyla and Genera

The 2,030,671 high-quality *nifH* reads were clustered into 1,274 OTUs at 95% amino acid identity. Differences in Shannon diversity showed no clear trend (Supplementary Figure 3). At the phylum level, the relative abundance of unidentified *nifH* accounted for 75% to 90% of total *nifH* gene abundances. Proteobacteria (7.9–23.7%) were the dominant identified phyla in all soils across the chronosequence (Supplementary Figure 4A). Among the 11 identified genera with an average relative abundance > 0.01%, the relative abundances of *Burkholderia* (3.1–11%), *Paraburkholderia* (1.6–16.3%), and *Geobacter* (0.6–4.3%) were > 0.5% across all sites (Supplementary Figure 4B). Based on both the weighted and unweighted UniFrac distances, | βNTI| was less than 2 in all sites, implying that the differences in phylogenetic composition resulted from stochastic processes (Supplementary Figure 5).

### Diazotrophic Community Composition, Variation, and Determinants

**FIGURE 2:**
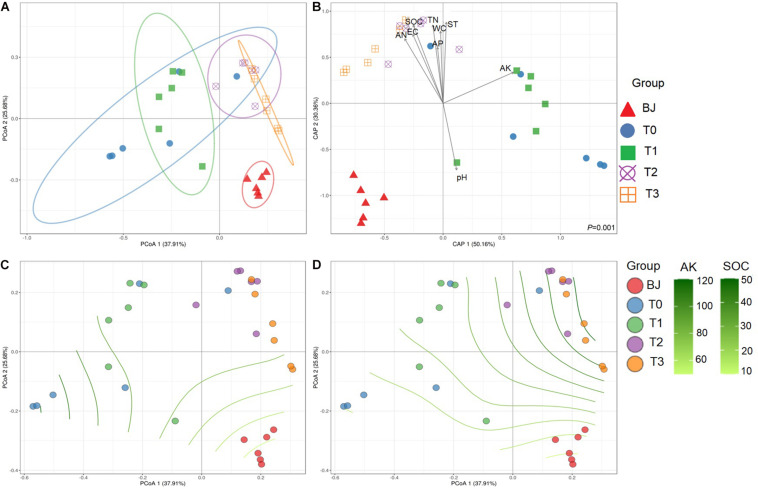
Beta diversity of the *nifH*-harboring bacterial communities and the relationships between communities and edaphic factors along the Hailuogou Glacier chronosequence. **(A)** Principal coordinate analysis based on Bray–Curtis dissimilarities between samples. **(B)** Canonical analysis of principal coordinates (CAP) using edaphic factors as explanatory variables and community Bray–Curtis dissimilarities as response variable. The non-linear regression analysis of AK **(C)** and SOC **(D)** based on distance matrix. Sample codes: BJ, successional age < 5 years; T0, successional age 22 years; T1, successional age 40 years; T2, successional age 54 years; T3, successional age 62 years. ST, soil temperature; EC, soil electrical conductivity; WC, soil gravimetric water; SOC, soil organic carbon; TN, total nitrogen; AN, available nitrogen; AP, available phosphorus; AK, available potassium.

In contrast to the separation based on soil properties in the PCA (Figure 1), the sites were not clearly separated based on the Bray–Curtis dissimilarities of the diazotrophic communities in the PCoA (Figure 2A). The diazotrophic communities from BJ were significantly dissimilar to those from the other sites. On sites T0 and T1, the within-site similarities were larger than on other sites, despite the relatively different within-site physicochemical characteristics. Consequently, the dissimilarities in community composition correlated with dissimilarities in edaphic factors according to both the CAP (Figure 2B) and the Mantel test (*P* < 0.01). According to the partial Mantel test, differences in AK and SOC correlated with differences in community composition between the sites (*P* < 0.01) (Supplementary Figure 6). Furthermore, the non-linear regression analysis of AK and SOC based on distance matrix showed the process of the effect of AK and SOC on the *nifH*-harboring bacterial community across the chronosequence (Figures 2C,D).

### Differentially Abundant Genera

**FIGURE 3:**
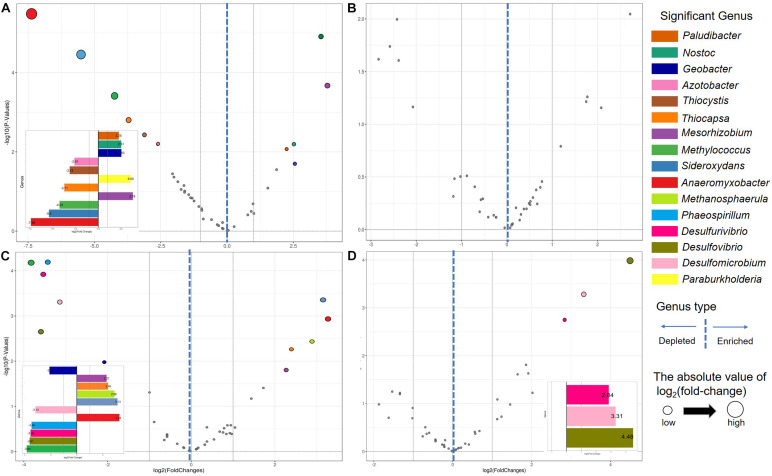
Volcano plots illustrating significantly enriched and depleted genera in the Hailuogou Glacier chronosequence. Each point represents an individual genus. **(A)** BJ (successional age < 5 years) vs. T0 (successional age 22 years). **(B)** T0 vs. T1 (successional age 40 years). **(C)** T1 vs. T2 (successional age 54 years). **(D)** T2 vs. T3 (successional age 62 years).

Genera characterizing differences between contiguous sites along the chronosequence were determined using differential abundance analysis (Supplementary Tables 4, 5). In total, five eGENs and six dGENs were detected between BJ and T0 (Figure 3A), five eGENs and six dGENs between T1 and T2 (Figure 3C), and three eGENs between T2 and T3 (Figure 3D). The relative abundances of most of the differentially abundant genera were low. For nine genera, the difference in relative abundances was detected in one comparison only. The relative abundances of *Anaeromyxobacter* and *Thiocapsa* were higher in BJ than in T0 and lower in T1 than in T2, and that of *Geobacter* was lower in BJ than in T0 and higher in T1 than in T2. The relative abundance of *Mesorhizobium* was lower in BJ than in T0 and lower in T1 than in T2, and that of *Methylococcus* was higher in BJ than in T0 and higher in T1 than in T2. The relative abundances of *Desulfomicrobium*, *Desulfurivibrio*, and *Desulfovibrio* were lower in T2 than in T3.

## Discussion

Biological transformation of N, the N cycle, is crucial in soil ecosystems. N fixation ability gives the diazotrophs and the interacting pioneer plants a selective advantage under N-limited conditions in developing soils (Duc et al., 2009). We studied N_2_-fixing communities carrying *nifH*, an essential gene in N fixation, in a glacier forefield chronosequence undergoing primary succession to assess succession-related changes in N_2_-fixing communities and to determine soil parameters associated with these changes.

During primary succession in the Hailuogou chronosequence, the coverage of plants and nutrient contents increased and soil pH decreased (Jiang et al., 2018; Bai et al., 2020). The increased plant coverage and the accompanying increased C input along the primary succession benefit N-fixing bacteria (Julien et al., 2011), possibly explaining the increase in copy numbers of the *nifH* gene and total DNA content along the chronosequence. In line with the assumption that bacterial N_2_ fixation is a major driver for the initial N input in non-fertilized soils (Julien et al., 2011), *nifH* copy numbers correlated positively with N contents and with the acetylene reduction rate, an indicator of nitrogen fixation activity, during the primary succession.

During primary succession, microbial communities may change irregularly and non-linearly, and the complex process includes changes in alpha and beta diversity from early to late successional stages within individual study (Ortiz-Álvarez et al., 2018). The bacterial communities change with soil depth even within the top 20 cm (Rime et al., 2015). Thus, studies with an approach similar to ours provide a community average within the topsoil. In a recent meta-analysis on primary succession, alpha diversity was expected to increase from early to late successional stages due to more heterogeneous habitats, more diverse niches and resources, and higher availability of resources; however, the changes in the alpha diversity of microbial communities from glacier forefield chonosequences showed no consistent trends (Ortiz-Álvarez et al., 2018). Interspecific competition for resources between plant and microorganism has been proposed to counterbalance the expected increase in alpha diversity (Schulz et al., 2013; Ortiz-Álvarez et al., 2018). In the Hailuogou Glacier forefield, the nutrient contents increased along the chronosequence (Bai et al., 2020). Thus, the increases in alpha diversity from T0 (successional age ≤ 22 years) to T2 (successional age ≈ 54 years) may have been partly due to less competition for resources. Even though primers, e.g., the *nifH* universal primers PolF and PolR, may not amplify all taxa with equal efficiency and thus the diversity estimates are likely biased (Ryan et al., 2016; Lee et al., 2019), the bias is not expected to affect largely within-study comparisons between sites.

In a meta-analysis on primary succession, the taxonomic compositions of late successional communities were more similar to one another than to those of early successional communities (Ortiz-Álvarez et al., 2018). In agreement, in the Hailuogou chronosequence, the within-site dissimilarities were larger on sites with successional ages of 20 and 40 years than on those with successional ages of 54 and 62 years, representing barren-developing and mature successional communities, respectively (Bai et al., 2020). The communities in the site with less than 4 years of successional age were seemingly uniform; possibly, taxa were missed below detection level due to the low abundance of *nifH* on the site.

In the Hailuogou chronosequence, the bacterial communities in the site with 3 years of successional age were different from the rest (Jiang et al., 2018), and the *phoD* communities in the sites with 20 and 40 years of successional age were different from those in the sites with 54 and 62 years of successional age (Bai et al., 2020). In our study, the diazotrophic communities in the site with less than 5 years of successional age were different from those in the site with 40 years of successional age. The discrepancy between the succession of bacterial communities and of *phoD* and the diazotrophic communities might have been due to functional redundancy: functional traits may be shared between different species (Louca et al., 2018). Due to functional redundancy, changes in the abundance of functional groups were proposed to capture the ecosystem function in heterogeneous ecosystems more accurately than changes in overall community composition (Kivlin and Hawkes, 2020). In agreement, the abundance of *nifH* correlated with soil N content, whereas no trend was apparent in the succession of the diazotrophic community composition. In soils with a wide pH range, pH had a bigger effect on the diazotrophic communities than other edaphic factors (Wang et al., 2017; Feng et al., 2018). In our study, possibly due to the narrow and close-to-neutral pH range, AK and SOC contents played bigger roles than pH on the changes in diazotroph community composition in the Hailuogou chronosequence.

The lack of a clear trend in the change in the diazotrophic community composition along the chronosequence was evident at the genus level as well. Among the differentially abundant genera, the relative abundances of *Geobacter* were lower in the beginning and at the end of the chronosequence than in the middle. The decrease toward the end may be connected to the anaerobic nature of *Geobacter* (Lovley et al., 2011); possibly, the increasing vegetation along the chronosequence resulted in more aerated soils that were not suitable for *Geobacter*. The relative abundances of *Mesorhizobium* spp. that fix N in symbiosis with legumes (Jarvis et al., 1997) increased along the chronosequence. In the Hailuogou chronosequence, *Astragalus* sp., a host plant of *Mesorhizobium*, was relatively dominant at sites ice free for approximately 26 to 45 years (Yang et al., 2014), which may have resulted in gradually higher relative abundances of *Mesorhizobium* from barren to developing stages. In a glacier forefield in Norway, the abundance of methanotrophs was higher with increasing distance from the glacier (Mateos-Rivera et al., 2018). Contrary to that, the relative abundance of *Methylococcus*, a methanotroph, was gradually lower from barren to developing stages. Since the *nifH* gene abundance became simultaneously higher, the observed lower relative abundances of *Methylococcus* may have been due to higher abundances of other *nifH*-carrying genera and not due to lower absolute abundances. As a methane-oxidizing bacterium, *Methylococcus* can use atmospheric CH_4_ to fix N even in carbon-poor environments (Duc et al., 2009; Kolb et al., 2010). Therefore, *Methylococcus* may have had competitive advantage over other diazotrophs at the carbon-poor barren stage, but the advantage was gradually lost with the increase in C input. Overall, the changes in plant coverage and species composition seem to be connected to the changes of diazotrophic communities during primary succession in the Hailuogou chronosequence.

Deterministic processes shaped the diazotrophic communities and βNTI correlated with pH and C and N contents in the alpine meadows of Qinghai-Tibet Plateau (Wang et al., 2017), whereas in fertilized agricultural soils, βNTI distribution was consistent with stochastic community assembly (Feng et al., 2018). In our study, the whole primary succession of the diazotrophic communities was consistent with stochastic community assembly, which is indicative of low competitive pressure (Dini-Andreote et al., 2015). Deterministic processes are considered to prevail in extreme and low-resource environments (Stegen et al., 2012). In the Hailuogou chronosequence, the pH values were close to neutral, and C content became gradually higher, which might explain the difference to the alpine meadows and similarity to the agricultural soils in community assembly.

In summary, the abundance of *nifH* became higher, and the composition of the diazotrophic communities changed during the primary succession in the Hailuogou chronosequence. The increased plant coverage and the accompanying increased C input along the primary succession possibly explained the increase in copy numbers of the *nifH* gene along the chronosequence. The changes in community composition lacked a clear trend and were mostly associated with changes in soil AK and SOC contents. The community assembly was stochastic, possibly due to low competitive pressure.

## Data Availability Statement

The datasets presented in this study can be found in online repositories. The names of the repository/repositories and accession number(s) can be found below: https://www.ncbi.nlm.nih.gov/, PRJNA511567.

## Author Contributions

YW: visualization and writing-original draft preparation. YC: investigation. QXu: visualization and investigation. QXi: software. KZ: software and validation. XY: data curation. QC: project administration. MM: formal analysis. HJ: resources. XZ: project administration. PP: project administration. YG: conceptualization, supervision, and funding acquisition. All authors contributed to the article and approved the submitted version.

## Conflict of Interest

The authors declare that the research was conducted in the absence of any commercial or financial relationships that could be construed as a potential conflict of interest.

## Footnotes

1 https://www.ncbi.nlm.nih.gov/sra/

2 http://qiime.sourceforge.net/
