# Supplementary material for: The Abundance of the nifH Gene Became Higher and the nifH-Containing Diazotrophic Bacterial Communities Changed During Primary Succession in the Hailuogou Glacier Chronosequence, China
Source: Front Microbiol. 2021 May 31;12:672656. doi: 10.3389/fmicb.2021.672656 (PMC8200853; doi:10.3389/fmicb.2021.672656)
Supplement: Supplementary file 1 [file Data_Sheet_1.docx]

| Sample code | Longitude  (E) | Latitude  (N) | Altitude  (m) | Successional age (years) | The distance from the glacier forefront (km) | Principal species |
| --- | --- | --- | --- | --- | --- | --- |
| BJ | 101°59′32.8″ | 29°34′03.6″ | 2956 | <5 | 0.1 | *Astragalus adsurgens, Hippophae rhamnoides, Salix magnifica, Populus purdomii* |
| T0 | 101°59′40.2″ | 29°34′04.1″ | 2948 | 22 | 0.5 | *Hippophae rhamnoides, Salix magnifica, Populus purdomii* |
|  |  |  |  |  |  |  |
| T1 | 101°59′43.7″ | 29°34′05.7″ | 2940 | 40 | 1.0 | *Hippophae rhamnoides, Salix magnifica, Populus purdomii, Betula utilis, Picea brachytyla* |
| T2 | 101°59′48.9″ | 29°34′07.9″ | 2926 | 54 | 1.3 | *Populus purdomii , Betula utilis, Picea brachytyla, Abies fabri* |
|  |  |  |  |  |  |  |
| T3 | 101°59′52.2″ | 29°34′10.9″ | 2919 | 62 | 1.5 | *Populus purdomii , Betula utilis, Picea brachytyla, Abies fabri* |
|  |  |  |  |  |  |  |

**Table S1 Basic information of** **the sampling sites along the Hailuogou Glacier chronosequence. Adopted from Bai et al (2020).**

**Table S2 Soil properties at the sampling sites along the Hailuogou Glacier chronosequence. Adopted from Bai et al (2020).**

|  | BJ | T0 | T1 | T2 | T3 |
| --- | --- | --- | --- | --- | --- |
| SOC (g▪kg^-1^) | 9.56±0.32d | 16.12±0.76c | 28.72±1.11b | 48.99±0.86a | 50.39±0.70a |
| TN (g▪kg^-1^) | 0.50±0.02d | 0.75±0.02c | 1.17±0.05b | 1.46±0.02a | 1.52±0.01a |
| AN (mg▪kg^-1^) | 8.32±0.61c | 15.25±1.04c | 42.19±3.05b | 56.47±5.10b | 96.43±5.16a |
| AP (mg▪kg^-1^) | 4.18±0.27d | 6.13±0.25d | 68.62±1.39a | 42.43±0.84b | 70.15±3.22a |
| AK (mg▪kg^-1^) | 45.78±3.15d | 122.28±2.52a | 75.52±0.99c | 80.87±0.48b | 84.50±0.92b |
| DNA content (μg·g^-1^dry soil) | 0.72±0.06b | 0.49± 0.03b | 0.70±0.04b | 2.90±0.12a | 2.44±0.07a |

Values (mean ± SE, *n* = 6) followed by different letters indicate statistically significant differences between sites (Tukey's HSD test, *p* < 0.05). EC: Soil electrical conductivity, WC: Soil gravimetric water, SOC: Soil organic carbon, TN: Total nitrogen, AN: Available nitrogen, AP: Available phosphorus, AK: Available potassium. BJ: successional age <5 years, T0: 22 years, T1: 40 years, T2: 54 years, T3: 62 years.

**Table S3 The Pearson correlation between acetylene reduction rate, *nifH* gene copy number and soil properties in the Hailuogou Glacier chronosequence.**

|  | ST | EC | pH | WC | SOC | TN | AN | AP | AK | DNA | ARA | *nifH* |
| --- | --- | --- | --- | --- | --- | --- | --- | --- | --- | --- | --- | --- |
| ARA | 0.892^**^ | 0.666^**^ | -0.790^**^ | 0.777^**^ | 0.714^**^ | 0.841^**^ | 0.700^**^ | 0.708^**^ | 0.677^**^ | 0.634^**^ | 1 | 0.425^*^ |
| *nifH* | 0.330 | 0.710^**^ | -0.520^**^ | 0.627^**^ | 0.788^**^ | 0.704^**^ | 0.743^**^ | 0.526^**^ | 0.196 | 0.552^**^ | 0.425^*^ | 1 |

*, p<0.05; **, p<0.01. ST: Soil temperature, EC: Electrical conductivity, WC: Soil gravimetric water, SOC: Soil organic carbon, TN: Total nitrogen, AN: Available nitrogen, AP: Available phosphorus, AK: Available potassium, ARA: Acetylene reduction rate, DNA: DNA content.
