# Supplementary material for: The Abundance of the nifH Gene Became Higher and the nifH-Containing Diazotrophic Bacterial Communities Changed During Primary Succession in the Hailuogou Glacier Chronosequence, China
Source: Front Microbiol. 2021 May 31;12:672656. doi: 10.3389/fmicb.2021.672656 (PMC8200853; doi:10.3389/fmicb.2021.672656)
Supplement: Supplementary file 4 [file Data_Sheet_4.docx]

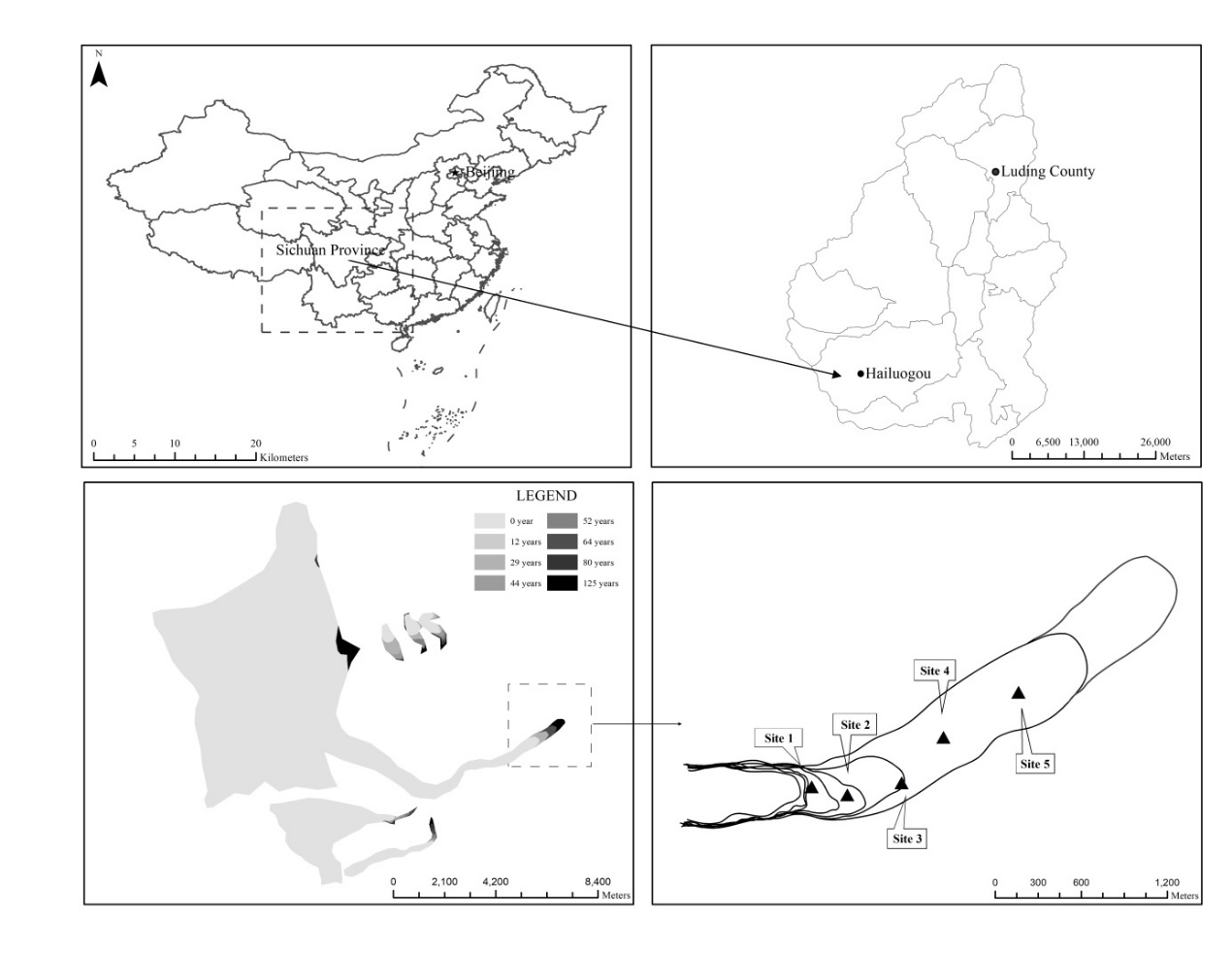


**Fig. S1** Location of the Hailuogou Glacier chronosequence and sampling sites. Site 1: BJ, successional age <5 years, Site 2: T0, 22 years, Site 3: T1, 40 years, Site 4: T2, 54 years, Site 5: T3, 62 years. Adopted from Bai et al (2020).

**Fig. S2. Abundances of *nifH* genes along the Hailuogou** **Glacier chronosequence**. BJ: successional age <5 years, T0: 22 years, T1: 40 years, T2: 54 years, T3: 62 years. Different letters above the bars indicate significant differences based on protected LSD test (p < 0.05).


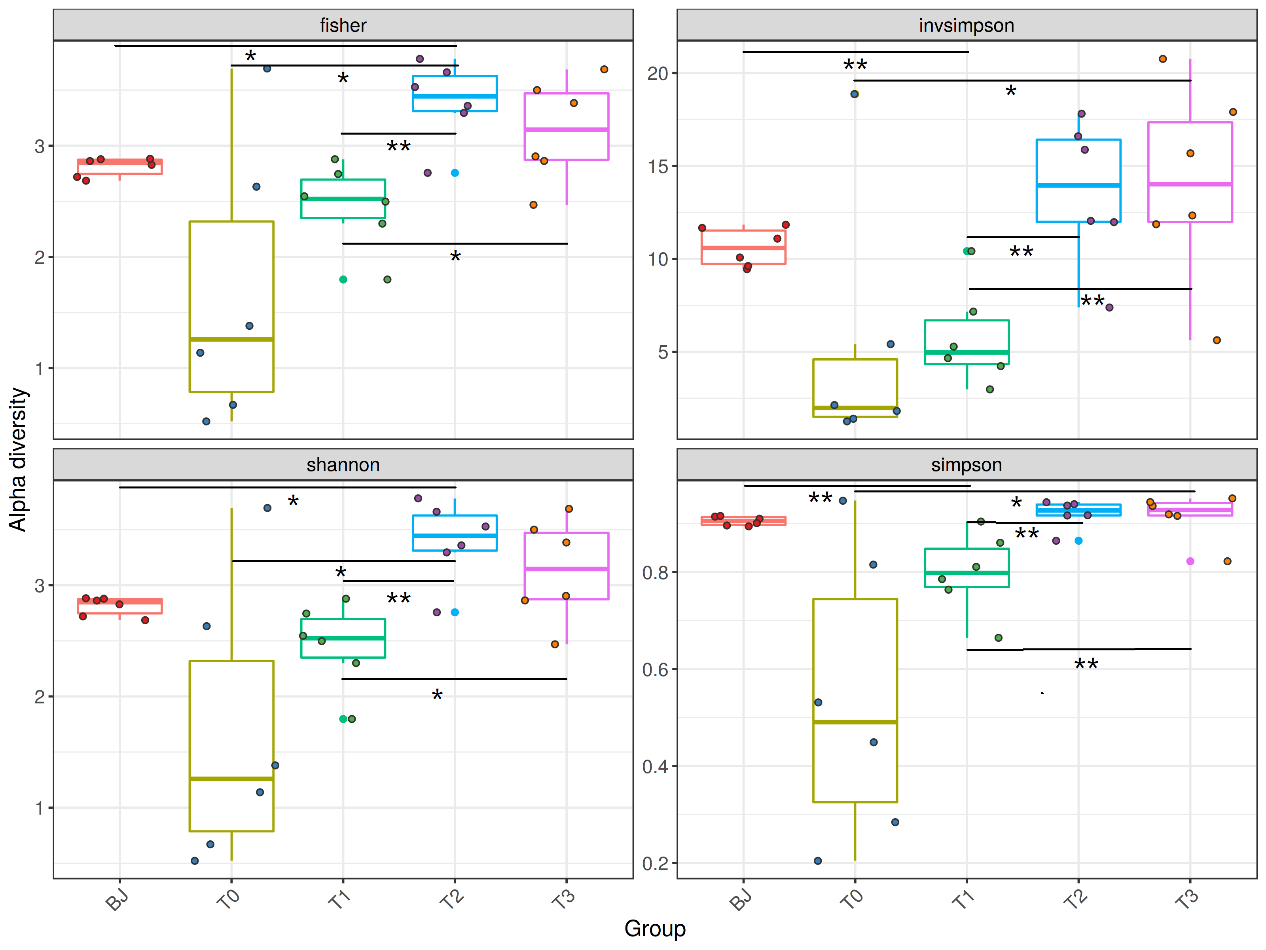


**Fig. S3 The diversity of the *nifH*-harboring bacterial communit****ies along the Hailuogou Glacier chronosequence.** The asterisks indicate statistically significant differences (* for *p* < 0.05, ** for *p* < 0.01, n = 6) between sites along the Hailuogou Glacier chronosequence. BJ: successional age <5 years, T0: 22 years, T1: 40 years, T2: 54 years, T3: 62 years.

**
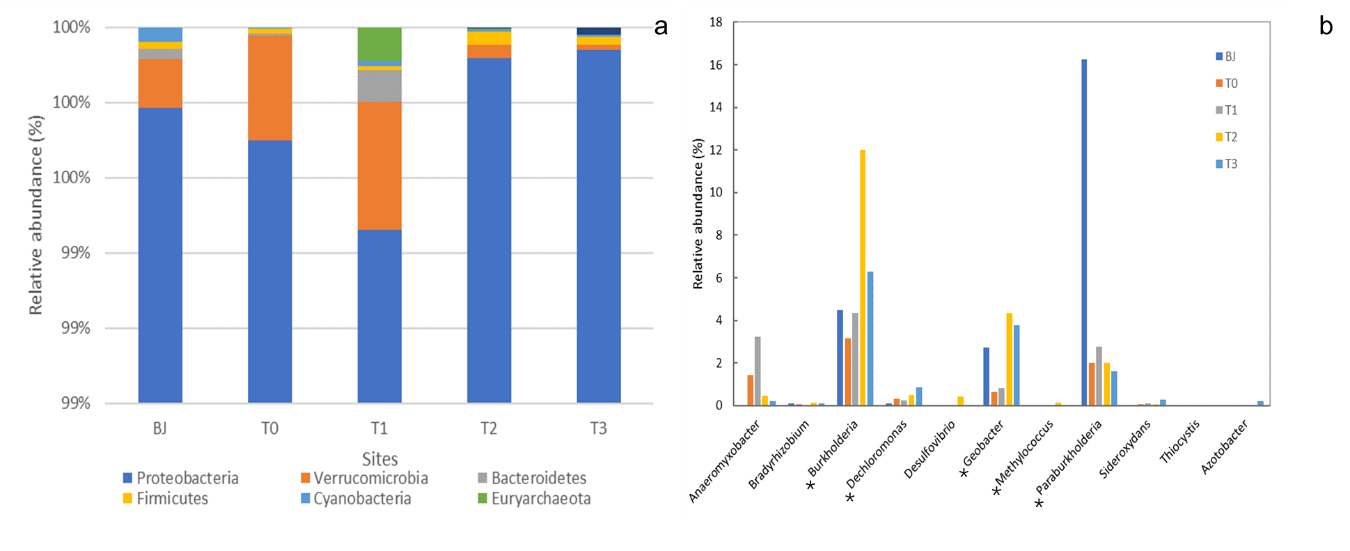
**

**Fig. S4 The relative abundance of (a) major *nifH*-harboring bacterial phyla and (b) 11 most abundant bacterial genera in** **the Hailuogou Glacier chronosequence.** BJ: successional age <5 years, T0: 22 years, T1: 40 years, T2: 54 years, T3: 62 years. * indicates statistically significant differences at *P* < 0.01.


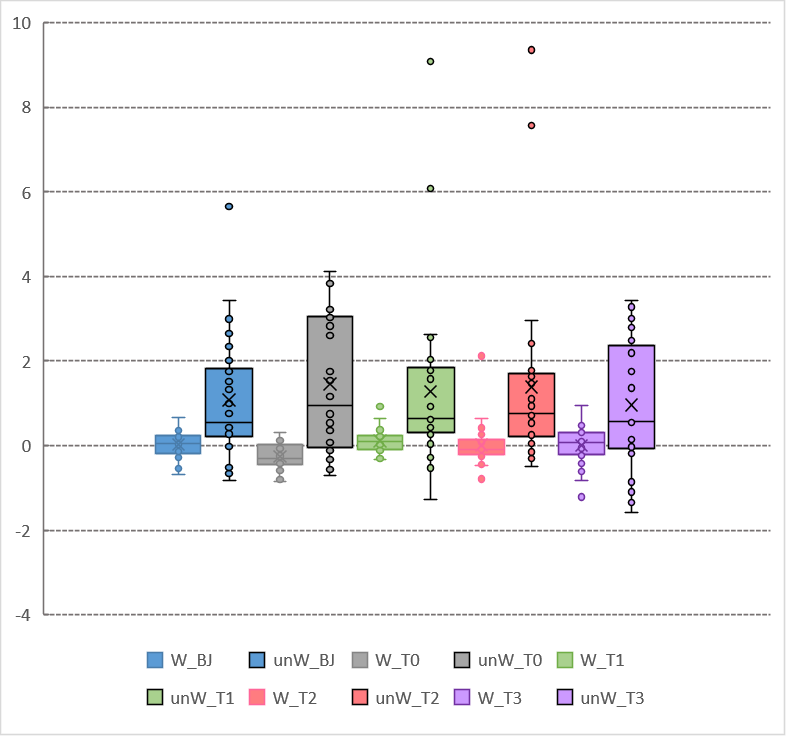


**Fig. S5 The β-nearest taxon index (βNTI) distributions of the *nifH*-harboring bacterial communities along the Hailuogou Glacier chronosequence.** Thick black line shows the median, the box shows the first and third quartile and open circles show the values more than 1.5 times the interquartile range. BJ: successional age <5 years, T0: 22 years, T1: 40 years, T2: 54 years, T3: 62 years. W, weighted Unifrac βNTI; unW, unweighted Unifrac βNTI.


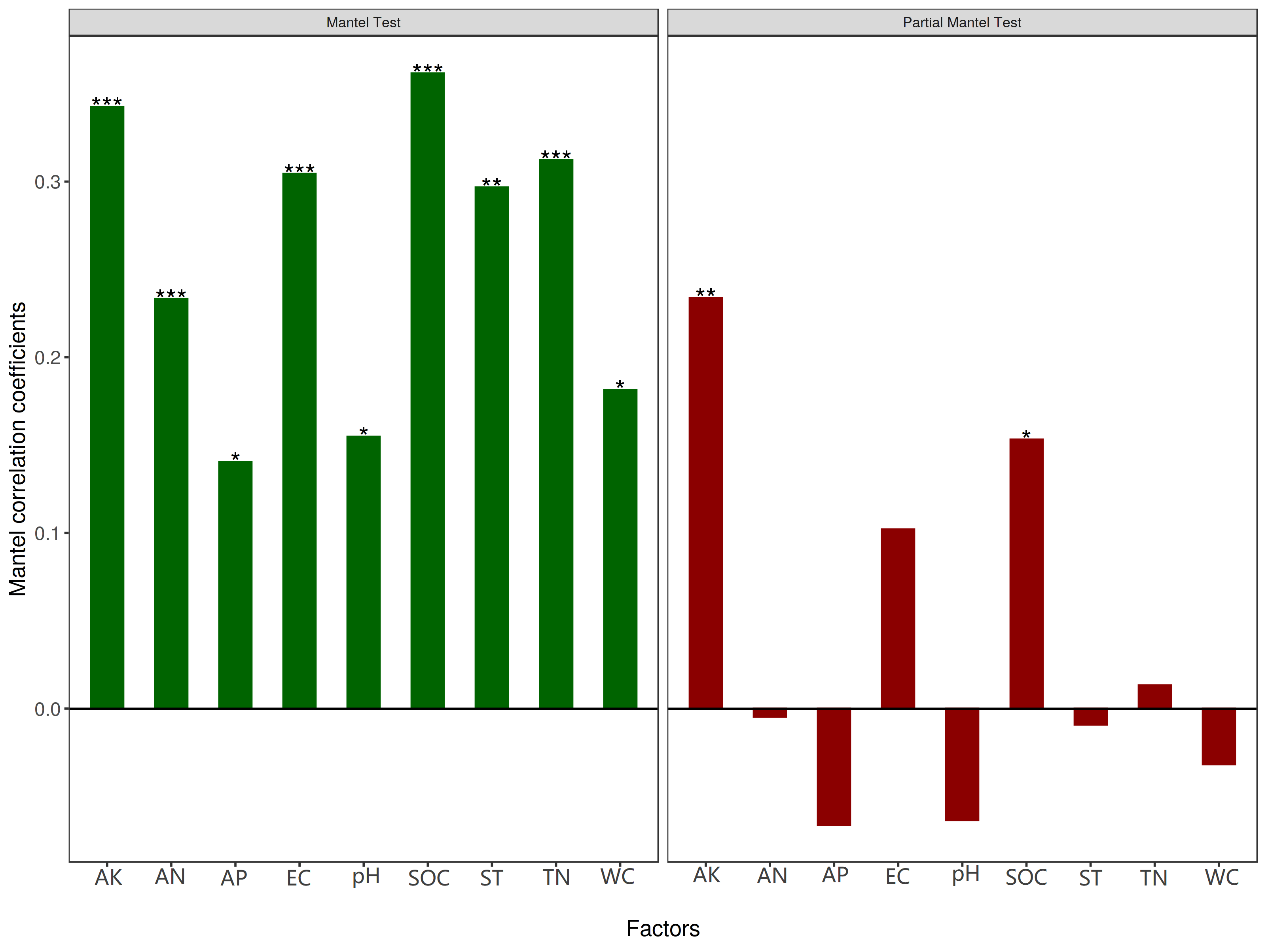


**Fig. S6 The relationships between edaphic properties and the *nifH*-harboring bacterial communities along the Hailuogou Glaicer chronosequence, based on Mantel test and Partial Mantel test.** ST: Soil temperature, EC: Soil electrical conductivity, WC: Soil gravimetric water, SOC: Soil organic carbon, TN: Total nitrogen, AN: Available nitrogen, AP: Available phosphorus, AK: Available potassium.
